# Exacerbation of Celecoxib-Induced Renal Injury by Concomitant Administration of Misoprostol in Rats

**DOI:** 10.1371/journal.pone.0089087

**Published:** 2014-02-21

**Authors:** Dustin L. Cooper, Derek E. Murrell, Christopher M. Conder, Victoria E. Palau, Grace E. Campbell, Shaun P. Lynch, James W. Denham, Angela V. Hanley, Kenny W. Bullins, Peter C. Panus, Krishna Singh, Sam Harirforoosh

**Affiliations:** 1 Department of Pharmaceutical Sciences, Gatton College of Pharmacy, East Tennessee State University, Johnson City, Tennessee, United States of America; 2 Gatton College of Pharmacy, East Tennessee State University, Johnson City, Tennessee, United States of America; 3 Department of Pathology, Quillen College of Medicine, East Tennessee State University, Johnson City, Tennessee, United States of America; 4 Department of Biomedical Sciences, Quillen College of Medicine, East Tennessee State University, Johnson City, Tennessee, United States of America; Emory University, United States of America

## Abstract

Nonsteroidal anti-inflammatory drugs (NSAIDs) can produce adverse effects by inhibiting prostaglandin (PG) synthesis. A PGE_1_ analogue, misoprostol, is often utilized to alleviate NSAID-related gastrointestinal side effects. This study examined the effect of misoprostol on celecoxib renal toxicity. Additionally, the effects of these drugs on cardiovascular parameters were evaluated. Four randomized rat groups were orally gavaged for 9 days, two groups receiving vehicle and two groups receiving misoprostol (100 µg/kg) twice daily. Celecoxib (40 mg/kg) was co-administered once daily to one vehicle and one misoprostol group from days 3 to 9. Urine and blood samples were collected and blood pressure parameters were measured during the study period. Hearts and kidneys were harvested on final day. Day 2 urinary electrolyte samples revealed significant reductions in sodium excretion in misoprostol (0.12±0.05 µmol/min/100 g) and misoprostol+celecoxib groups (0.07±0.02 µmol/min/100 g). At day 3, all treatment groups showed significantly reduced sodium excretion. Potassium excretion diminished significantly in vehicle+celecoxib and misoprostol+celecoxib groups from day 3 onward. Urinary kidney injury molecule-1 levels were significantly increased in vehicle+celecoxib (0.65±0.02 vs. 0.35±0.07 ng/mL, p = 0.0002) and misoprostol+celecoxib (0.61±0.06 vs. 0.37±0.06 ng/mL, p = 0.0015) groups when compared to baseline; while plasma levels of cardiac troponin I increased significantly in vehicle+celecoxib (p = 0.0040) and misoprostol+misoprostol (p = 0.0078) groups when compared to vehicle+vehicle. Blood pressure parameters increased significantly in all misoprostol treated groups. Significant elevation in diastolic (p = 0.0071) and mean blood pressure (p = 0.0153) was noted in misoprostol+celecoxib compared to vehicle+celecoxib. All treatments produced significant tubular dilatation/necrosis compared to control. No significant myocardial changes were noticed; however, three animals presented with pericarditis. Kidney, heart, and plasma celecoxib levels revealed no significant change between vehicle+celecoxib and misoprostol+celecoxib. Concomitant misoprostol administration did not prevent celecoxib renal toxicity, and instead exacerbated renal side effects. Misoprostol did not alter plasma or tissue celecoxib concentrations suggesting no pharmacokinetic interaction between celecoxib and misoprostol.

## Introduction

Nonsteroidal anti-inflammatory drugs (NSAIDs) are highly efficient drugs used in a variety of diseases because of their anti-inflammatory, antipyretic, and analgesic effects. NSAIDs function through the inhibition of cyclooxygenase (COX), an enzyme necessary for prostaglandin (PG) formation. NSAIDs are categorized based on their specific mechanism of action. Non-selective NSAIDs, such as diclofenac and indomethacin, function to inhibit both COX-1 and COX-2 enzymes; while COX-2-selective inhibitors (COXIBs), such as celecoxib and rofecoxib, function to inhibit only the COX-2 enzyme [1], [2].

Because of the highly efficient nature of NSAIDs, a large number of adverse gastrointestinal (GI) and renal side effects are associated with their usage. The most prevalent GI side effects include stomach bleeding, indigestion, and ulceration. Edema and electrolyte retention are the predominant NSAID-related renal side effects [3]–[7]. It has also been found that individuals who present with cardiovascular (CV) complications may be at an increased risk of developing myocardial infarction (MI) or stroke, when undergoing prolonged NSAID therapy [8]–[11].

NSAIDs can produce toxic effects in both the GI and renal systems. Furthermore, several studies have established a link between COXIBs and CV adverse events [12]. COXIBs (e.g. celecoxib) were found to cause sodium retention by increasing the expression of Na^+^/K^+^/2Cl^−^ cotransporter (NKCC2) in renal tubules. Another transporter heavily involved in sodium regulation, Na^+^/K^+^-ATPase (NKA), may fluctuate with changing PG levels contributing to sodium retention [13], [14]. A decrease in urinary sodium excretion has been shown to be associated with an increased risk of CV events [15], [16]. In a systematic review conducted by McGettigan and Henry, celecoxib was found to confer an overall increase in CV risk with doses exceeding 400 mg/day [12]. This dose-dependent relationship between celecoxib and CV risk was also uncovered in a meta-analysis by Solomon *et al.* which exhibited an increase in the relative risk of CV events as the daily dose of celecoxib increased from 400–800 mg [11].

Misoprostol is a synthetic analogue of PGE_1_ that has gained considerable attention as a powerful reactive oxygen species scavenger [17] showing strong anti-apoptotic and cytoprotective effects [18]. Over the years, misoprostol use has been successful in the treatment of liver cell necrosis and intestinal cell apoptosis and has been approved by the FDA for the treatment of NSAID-related GI side effects [17], [19]–[22]. The gastroprotective effects of misoprostol were first described by Robert *et al.* in 1967, and have become the active standard against which newer gastroprotective interventions are tested. As such, misoprostol is commonly used for the prevention of deleterious GI side effects of NSAIDs such as diclofenac (a non-selective NSAID) [19], [23], [24].

Misoprostol has also been found to affect the expression of NKCC2 [25] through cyclic adenosine monophosphate (cAMP) regulation. NSAIDs consumption reduces PGE_2_ levels through COX inhibition, which decreases cAMP expression, allowing for increased NKCC2 expression. Misoprostol has been shown to reverse the stimulatory effects of NSAIDs on NKCC2 expression [26].

The objective of this study was to evaluate the effect of misoprostol on celecoxib-induced renal toxicity. Furthermore, the CV effects of celecoxib alone or in combination with misoprostol were examined.

## Materials and Methods

### Chemicals

High performance liquid chromatography (HPLC) grade chemicals (iso-octane, 2-propanol, acetonitrile, water, acetic acid, sulfuric acid, and triethanolamine) were purchased from Fischer Scientific Laboratory (Fair Lawn, NJ, USA). Celecoxib was purchased from Toronto Research Chemicals, Inc. (North York, ON, Canada); while Methylcellulose 4000 was purchased from Science Stuff, Inc. (Austin, TX, USA). Misoprostol was purchased from the Cayman Chemical Company (Ann Arbor, MI, USA) and ibuprofen was obtained from Sigma-Aldrich (St. Louis, MO, USA).

### Animals and Drug Administration

In accordance with guidelines established by the University Committee on Animal Care (UCAC) at East Tennessee State University, experimental procedures were carried out on male Sprague-Dawley rats, ranging in weight from 240 to 290 g, following an UCAC reviewed and approved protocol (#P110901). GPower 3.1, a software program which determines statistical power, was used to identify an appropriate study sample size. The effect size based on a previous study, which revealed a 65±25% (mean ± standard deviation) sodium excretion decrease in rats treated with celecoxib, was 40% [27]. Using an effect size of 0.4 and a power of 95%, the detection of a significant difference (p<0.05) in sodium excretion rate required a sample size of 24. Thus this study consisted of 4 groups (n = 6).

Celecoxib or misoprostol was dissolved in a 0.5% methylcellulose solution and administered via gastric intubation. A significant change in electrolyte excretion has been previously reported with a celecoxib dose of 40 mg/kg/day [28]. Therefore, in the present study, animals received the same drug dosage. Kurtz *et al.* have demonstrated an increase in blood pressure one week following treatment with a COXIB (rofecoxib) in normotensive rats [29]. Therefore, we administered celecoxib for one week. The dosage of misoprostol (200 µg/kg/day) was chosen based on data published by Ozer *et al.* [30].

### The Study Design

On day 0, rats were divided into 4 groups (n = 6). On days 1 and 2, vehicle+vehicle (control) and vehicle+celecoxib groups were dosed with vehicle (methylcellulose solution) twice daily. The misoprostol+misoprostol and misoprostol+celecoxib groups were dosed with misoprostol (100 µg/kg) twice daily. On days 3–9, the vehicle+vehicle group received vehicle twice daily. The vehicle+celecoxib group received a single daily dose of celecoxib (40 mg/kg) in the morning and vehicle once daily in the afternoon. The misoprostol+misoprostol group received misoprostol (100 µg/kg) twice daily. The misoprostol+celecoxib group received misoprostol (100 µg/kg) twice daily along with a single daily dose of celecoxib (40 mg/kg). On days 0, 2, 3, and 9, animals were transferred to metabolic cages and housed 8 hours (from 8 a.m. to 4 p.m.) for urine sample collection. Following urine collection, blood samples (500 µL) were taken via the tail clip method into a capillary blood collection tube containing lithium heparin. The tube was then centrifuged at 12,000 rpm for 3 minutes. The supernatant was transferred to a microcentrifuge tube and kept at −80°C until analysis. During the study, blood pressure measurements were obtained by using a two channel, non-invasive, tail-cuff blood pressure monitoring system (Kent Scientific, Torrington, CT). On day 10 under anesthesia, blood was collected via heart puncture through the diaphragm. The heart, liver, and kidneys were excised, snap frozen, and stored at −80°C for analysis.

### Electrolyte Analysis

The levels of sodium and potassium in plasma or urine were determined using an EasyLyte analyzer (Medica Corporation, Bedford, MA, USA). Urinary ion excretion rates were determined using an equation, C_×_V_×_100/T_×_W, which involves the detected concentration (mmol/L) of Na^+^ or K^+^ in the urine sample, the total urine volume in milliliters (V), collection time in minutes (T), and the body weight of the animal in grams (W).

### Determination of Urinary Kidney Injury Molecule-1 (KIM-1) Concentrations

Urinary KIM-1 levels were assessed using an enzyme-linked immunosorbent assay (ELISA) in accordance with manufacturer instructions (Rat KIM-1 ELISA Test Kit, Kamiya Biomedical Company, Seattle, WA, USA) [31], [32]. The frozen urine samples were thawed; allowed to come to room temperature; and then diluted 1∶3 with the provided dilution buffer. Standard calibrators were serially diluted from 10 to 0.313 ng/mL. In a micro-titer plate, 50 µL of dilution buffer and 25 µL of corresponding sample (calibrator or urine) were added to each well. Samples were allowed to equilibrate on a rotary shaker for one minute. Fifty microliters of each sample was then quickly transferred to corresponding ELISA plate wells containing 50 µL of blocker/stabilizer solution. Samples were incubated for 120 minutes and then washed for 10 seconds, after which 100 µL of anti-KIM-1 Conjugate was added to each well. Further incubation (60 minutes) and then another wash were performed. One hundred microliters of substrate solution was then added to the wells and incubated for another 20 minutes. After incubation, 100 µL of stop solution was added to each well. The plate was read at 405 nm with a 490 nm differential filter. Curve-fitting was performed using the 3^rd^ order polynomial regression of the cloud-based data analysis software (MyAssays Ltd, Sussex, England).

### Determination of Aldosterone Levels

Plasma aldosterone levels were measured using an aldosterone enzyme immunoassay (BioVendor, Asheville, NC, USA). Frozen plasma samples were thawed at room temperature. Then 50 µL of calibrator solution, control solution, and plasma samples were pipetted into correspondingly labelled wells in duplicate. Afterward, 100 µL of conjugate working solution was pipetted into each well using a multichannel pipette. The plate was then incubated at room temperature on a plate shaker set at 200 rpm for 1 hr. Following incubation, each well was washed 3 times with 300 µL of diluted wash buffer. After washing, 150 µL of TMB substrate was pipetted into each well and incubated for 20 minutes, after which 50 µL stop solution was added. The plate was immediately read at 450 nm. Cloud-based data analysis software was used to generate a standard curve using an exponential fit.

### Determination of Blood Urea Nitrogen (BUN)

Measurement of circulating BUN was carried out using a BUN enzymatic assay kit (Bioo Scientific Corporation, Austin, TX, USA). Briefly, 5 µL of plasma was added, in duplicate, to designated microwell plates, followed by 150 µL of urease mix solution. Solutions were then incubated for 15 minutes at room temperature. Following this, 150 µL of alkaline hypochlorite was added to each well then incubated again for another 10 minutes. After incubation, the plate was read at 620 nm.

### Determination of Plasma Levels of Cardiac Troponin I (cTnI)

To detect any heart injury during this study, plasma cTnI levels were measured based on a previously described method [33] using a K-ASSAY Rat Cardiac Tropinin-1 ELISA (Kamiya Biomedical Company, Seattle, WA, USA). Prior to assay, plasma samples were thawed and diluted 1∶4 with the provided plasma diluent. Lyophilized cTnI stock was reconstituted with 400 µL de-ionized water and gently mixed over 10 minutes. Calibrators were prepared using serial dilution (10, 5, 2.5, 1.25, 0.625, 0.312, 0.156 ng/mL). Plasma samples and calibrators were used within 30 minutes of preparation. One hundred microliters of cTnI HRP conjugate was added to each well followed by either 100 µL of calibrator or diluted sample. The plate was placed on a shaker (150 rpm) at room temperature for one hour. Plate contents were emptied and the micro-titer wells were rinsed six times with 1X wash solution. Residual droplets of wash solution were removed by striking the plate on a paper towel. Following the addition of 100 µL of tetramethylbenzidine to each well, the plate was placed on a plate shaker (150 rpm) for 20 minutes at room temperature. Stop solution (100 µL) was then added to each well and mixed gently. The plate was read at 450 nm.

### Blood Pressure Measurements

Using the CODA Standard Non-Invasive Blood Pressure System (Kent Scientific, Torrington, CT, USA), blood pressure parameters were obtained using a tail-cuff method described by Whitesall *et al.* [34]. Briefly, the rat was placed in a box restraint. A tail-cuff occluder was placed on the tail which passed through an optical sensor. The cuff was inflated then deflated slowly creating an occlusion followed by reperfusion detected by the sensor. The instrument was controlled by software which used measurements from a series of inflation-deflation cycles to calculate blood pressure.

### Histopathological Assessments

Kidney and heart samples were placed in 10% formalin for 24 hours. Thick sections (0.5 µm) were prepared for each heart and each kidney then stained using hematoxylin and eosin stain (H&E). Each slide was reviewed by a board certified anatomic and clinical pathologist who was unaware of treatment groups.

The kidney was evaluated based on tubular necrosis, tubular dilatation, and glomeruli sparing. Tubular necrosis was graded on a scale from 0 to 3 based on the geographic area and extent of necrotic tubules (0 = normal/no tubular necrosis, 1 = focal area of tubular necrosis involving less than 10% of the kidney, 2 = tubular necrosis involving 10–25% of the kidney, 3 = tubular necrosis involving greater than 25% of the kidney). Tubular dilatation was graded on a scale of 0 to 3 (0 = normal tubules, 1 = mild dilatation, 2 = moderate dilatation, 3 = severe dilatation). Glomeruli were examined for structural changes or damage.

The heart was evaluated for any abnormalities of the myocardium (inflammation, infarct, and/or scarring).

### TUNEL Assay

Slides were assayed for apoptosis using an *In Situ* Cell Death Detection Kit, Fluorescein following manufacturer instructions (Roche Applied Science, Mannheim, Germany). Briefly, sections were isolated using a pap pen and washed with PBS. The tissue was incubated in permeabilizing solution (0.01% Triton-X in 0.01% Sodium Citrate) at room temperature for 8 minutes. Following PBS washing, 50 mL assay solution was added to each section then incubated at 37°C for one hour. The slides were washed with PBS then stained with DAPI for five minutes at room temperature. Following a final PBS wash, the slides were partially dried. Coverslips were applied to each slide following application of ProLong® Gold Antifade Reagent (Life Technologies, Grand Island, NY). Samples were allowed to dry then fluorescent images (20x magnification) were captured using an EVOS fluorescent microscope (AMG, Bothell, WA).

Images were quantified using apoptotic cell percentage. Six slide-representative sections (3 containing tubules and 3 containing glomeruli) were counted (nuclei and TUNEL positive) for each animal.

### Immunohistochemistry

#### Slide preparation

Paraffin-embedded kidney sections (4 µm) were prepared for TUNEL assay and immunohistochemistry. Slides were deparaffinized using xylene, graded ethanol concentrations (100, 95, 70, 50, and 30%), and double distilled water then incubated in PBS.

#### Immunohistochemical analysis, caspase-3

Slides were incubated in peroxo-block for 2 minutes at room temperature then washed twice for two minutes in PBS. The slides were incubated in 5% goat serum for 15 minutes then rabbit anti-caspase-3 (1∶20, dilution in 5% goat serum) overnight at 4°C. Following overnight incubation, the slides were washed for five minutes in PBS three times. The slides were then incubated in biotinylated secondary antibody (1∶1000, dilution in PBS) for 10 minutes; washed in PBS three times for 2 minutes; then incubated in streptavidin-peroxidase conjugate for 10 minutes. The slides were then washed twice for two minutes in PBS. AEC substrate was added and monitored for intensity. Upon completion, the reaction was stopped with dH2O (pH 8). Hematoxylin counterstain was added for 3 minutes then washed with PBS for 5 minutes. The slides were rinsed with dH2O (pH 8). GVA was added to the slide then coverslipped and sealed with Cytoseal. Slides were photographed using a Spot insight 4 camera and software (Leica Leitz Labor-Lux S, 40x objective).

#### Immunohistochemical analysis, NKCC2

Slides were incubated in peroxo-block for 2 minutes at room temperature then washed twice for two minutes in PBS. The slides were incubated in 5% goat serum for 15 minutes then primary antibody, rabbit anti-SLC12A1 N-term (GeneTex, Irvine, CA, USA; 1∶500 dilution in 5% goat serum) overnight at 4°C. Following overnight incubation, the slides were washed for five minutes in PBS three times. The slides were then incubated in biotinylated secondary antibody (1∶1000, dilution in PBS) for 10 minutes; washed in PBS three times for 2 minutes; then incubated in streptavidin-peroxidase conjugate for 10 minutes. The slides were then washed twice for two minutes in PBS. AEC substrate was added and monitored for intensity. Upon completion, the reaction was stopped with dH2O (pH 8). Hematoxylin counterstain was added for 3 minutes then washed with PBS for 5 minutes. The slides were rinsed with dH2O (pH 8). GVA was added to the slide then coverslipped and sealed with Cytoseal. Slides were photographed using a Spot insight 4 camera and software (Leica Leitz Labor-Lux S, 40x objective).

### Kidney Tissue Preparation and Immunoblotting

#### Kidney tissue preparation

NKCC2 and NKA abundance were measured in the kidney using a modified method published by Fernandez-Llama *et al.* [26] with major changes. One half of a kidney was placed in a 1.5 mL eppendorf tube then placed on ice. After 10 minutes, 1 mL of isolation buffer consisting of 250 millimolar (mM) sucrose, 10 mM triethanolamine, and Halt™ Protease and Phosphatase Inhibitor Single-Use Cocktail (Thermo Scientific, Rockford, IL, USA) was added to each tube. Samples were then homogenized at high speed using a Power Gen 125 electric homogenizer (Fischer Scientific, Pittsburgh, PA, USA) for 40 seconds on ice. Following homogenization, the samples were centrifuged using a cell sifter. Protein concentrations were then determined using a Pierce BCA Assay kit (Thermo Fischer, Rockford, IL, USA).

#### NKCC2 immunoblotting

Samples were diluted to a 1.25 µg/µL protein concentration using 2X laemelli sample buffer. Samples were vortex mixed, spun down briefly, and then heated at 60 degrees for 15 minutes. Following removal from heat, samples were maintained at room temperature for 5 minutes then spun down. Five microliters of precision plus pre-stained standard (Bio-Rad Laboratories, Hercules, CA, USA) and 20 µL of each sample were loaded into a Tris-Acetate 3–8% gel (Life Technologies, Carlsbad, CA, USA). The gel was electrophoresed at 150 volts for 1 hour in 1X Tris-acetate running buffer containing 500 µL of NuPage antioxidant (Invitrogen, Grand Island, NY, USA). The proteins were then transferred on to a nitrocellulose membrane at 40 volts for 2 hours. Following transfer, the membrane was stained with amido black for 5 minutes to visualize protein. Following destaining with DI H_2_O, the membrane was incubated with 2.5% BSA in phosphate buffered saline with Tween-20 (PBST) for 1 hour.

The nitrocellulose membrane was then cut into two pieces (top and bottom); top one containing the NKCC2 band (161 kDa) and bottom one containing β-actin (42 kDa). The top portion was probed with rabbit polyclonal primary antibody against NKCC2 (GeneTex, Irvine, CA, USA; 1∶400 dilution in PBST); while the bottom portion was probed with goat polyclonal primary antibody against β-actin (Santa Cruz Biotechnology, Dallas, TX, USA; 1∶500 dilation in PBST). After overnight incubation at 4°C, the blots were rinsed 3 times with PBST for 10 minutes. The first blot was then probed with goat anti-rabbit HRP secondary antibody 1∶5000 (Thermo Scientific, Rockford, IL, USA) and the second blot was probed with Peroxidase-AffiniPure donkey anti-goat secondary antibody 1∶5000 (Jackson ImmunoResearch Laboratories, Inc., West Grove, PA, USA) for 3 hours at 4°C. Following secondary antibody probing, the blots were rinsed 3 times for 10 minutes with PBST.

Following the final PBST wash, ECL SuperSignal West Pico Chemiluminescent Substrate (Thermo Scientific, Rockford, IL, USA) was added to the membrane and immediately placed in a G-Box Imager (Syngene, Fredrick, MD, USA) for detection. The images were analyzed using NIH software. NKCC2 band intensities were normalized to β-Actin band intensities to normalize for possible protein loading fluctuations.

#### NKA Immunoblotting

Samples were diluted to a 1.25 µg/µL protein concentration using 2X Laemelli sample buffer. Samples were vortex mixed, spun down briefly, and then heated at 60 degrees for 15 minutes. Following removal from heat, samples were maintained at room temperature for 5 minutes then spun down. Three microliters of precision plus pre-stained standard (Bio-Rad Laboratories, Hercules, CA, USA) and 20 µL of each sample were loaded into a Tris-HEPES-SDS gel (Thermo Scientific Pierce, Rockford, IL, USA). The gel was electrophoresed at 100 volts for 1 hour in 1X HEPES running buffer. The proteins were then transferred on to a nitrocellulose membrane at 30 volts for 2 hours. Following transfer, the membrane was stained with amido black for 5 minutes to visualize protein. Following destaining with DI H_2_O, the membrane was incubated with 1% BSA in phosphate buffered saline with Tween-20 (PBST) for 1 hour.

The nitrocellulose membrane was then cut into two pieces (top and bottom); top one containing the N-K-ATPase band (≈100 kDa) and bottom one containing β-actin (42 kDa). The top portion was probed with rabbit polyclonal primary antibody against NKA α-1 (GeneTex, Irvine, CA, USA; 1∶500 dilution in 1% BSA); while the bottom portion was probed with goat polyclonal primary antibody against β-Actin (Santa Cruz Biotechnology, Dallas, TX, USA; 1∶500 dilation in 1% BSA). After an overnight incubation at 4°C, the blots were rinsed 3 times with PBST for 10 minutes. The first blot was then probed with goat anti-rabbit HRP secondary antibody 1∶5000 (Thermo Scientific, Rockford, IL, USA) and the second blot was probed with Peroxidase-AffiniPure donkey anti-goat secondary antibody 1∶5000 (Jackson ImmunoResearch Laboratories, Inc., West Grove, PA, USA) for 3 hours at 4°C. Following secondary antibody probing, the blots were rinsed 3 times for 10 minutes with PBST.

Following the final PBST wash, ECL SuperSignal West Pico Chemiluminescent Substrate (Thermo Scientific, Rockford, IL, USA) was added to the membrane and immediately placed in a G-Box Imager (Syngene, Fredrick, MD, USA) for detection. The images were analyzed using NIH software. NKA band intensities were normalized to β-Actin band intensities to normalize for possible protein loading fluctuations.

### Chromatographic Conditions

#### Assay solution preparation and equipment

Prior to usage, the celecoxib extraction mobile phase, composed of acetonitrile, water, acetic acid, and triethanolamine (47∶53: 0.1∶0.03), was filtered using a 0.5 µm nylon filter. Serial dilutions (25–100,000 ng/ml) of stock celecoxib solution (10 mg in 100 mL mobile phase) were used to create a standard concentration curve. An internal standard was prepared by dissolving ibuprofen (10 mg) in 100 mL mobile phase. Cardiac and renal samples were homogenized using a Power Gen 125 electric homogenizer (Fischer Scientific, Pittsburgh, PA, USA). Sample organic phases were evaporated using a CentriVap concentrator (Lab Conoco, Kansas City, MO, USA). A HPLC system (Shimadzu, Japan) consisting of a LC020AB solvent delivery system, a SIL-20A HT auto-sampler with a SPD-M20A photodiode array detector set at 254 nm, a CBM-20A communication bus, a DGU-20A3 vacuum degasser, and a CTO-20A column oven containing a C18 analytical column (100×4.6 mm, 2.6 µm; Phenomenex, Torrance, CA, USA) was used to determine drug concentrations.

#### Plasma celecoxib extraction

For plasma extraction, 100 µL of standard celecoxib concentrations (25–100,000- ng/mL) was added to 100 µL blank rat plasma. After which, 200 µL sulfuric acid (0.6 M), 100 µL of internal standard, and 5 mL of iso-octane 2-propanol (95∶5) was added to each standard sample. Each solution was vortex mixed for 30 seconds and then centrifuged for 5 minutes at 2,500 g. Following centrifugation, the aqueous phase was frozen using a dry ice/ethanol bath. Each liquid organic phase was then transferred to a clean tube for evaporation. The samples were reconstituted using 200 µL mobile phase. Samples (100 µL each) were injected into the HPLC system and ran for 15 minutes using a mobile phase flow rate of 1 mL/min. The minimal detectable concentration of celecoxib in plasma was 25 ng/mL with a coefficient of variance of 4.2%.

#### Renal celecoxib extraction

Following removal from −80°C and subsequent thawing at room temperature, kidney samples (one half of each kidney) were homogenized in HPLC grade water (2∶1 water, sample weight ratio). For the calibration curve, blank kidney homogenate (100 µL) was added to clean glass tubes then spiked with 100 µL of standard celecoxib concentrations (25–100,000 ng/mL). Two hundred microliters of 0.6 M sulfuric acid, 100 µL internal standard, and 5 mL iso-octane 2-propanol (95∶5) were then added to each sample. Samples were vortex mixed for 30 seconds followed by centrifugation at 2,500 g for 5 minutes. A dry ice/ethanol bath was then used to freeze each aqueous phase which allowed the removal of each organic phase to a new glass tube. Samples were evaporated to dryness then reconstituted with 200 µL mobile phase. Samples (100 µL each) were injected into the HPLC. Each sample was ran at a mobile phase flow rate of 1 mL/min for 15 minutes. The minimum detectable concentration of celecoxib was 100 ng/g in kidney homogenates (coefficient of variance = 16.2%).

#### Cardiac celecoxib extraction

Heart samples (one half of each heart) were removed from −80°C and thawed at room temperature. Each sample was transferred to a clean tube, weighed, and homogenized in a 2∶1 HPLC grade water to sample weight ratio. One hundred microliters of blank heart homogenate was added to 100 µL of each standard celecoxib dilution (25–100,000 ng/mL). Two hundred microliters sulfuric acid (0.6 M) was added to each sample followed by 100 µL internal standard. After adding 5 mL of iso-octane 2-propanol (95∶5), samples were vortexed 30 seconds and centrifuged for 5 minutes at 2,500 g. Samples were then immersed in a dry ice/ethanol bath. The organic phase was transferred to a clean glass tube and evaporated to dryness. After which, samples were reconstituted in 200 µL mobile phase and vortex mixed. Samples (100 µL each) were injected into the HPLC and ran for 15 minutes with a flow rate of 1 mL/min. The minimum detectable concentration of cardiac celecoxib was 100 ng/g exhibiting a coefficient of variance of 0.1%.

### Data Treatment and Statistical Analysis

For the urinary electrolyte excretion rates, KIM-1 concentrations, and cTnI levels, data comparisons among the groups were made by two-way ANOVA, using the PROC MIXED procedure of SAS (SAS Institute Inc., Cary, NC, USA). For weight parameters, plasma electrolyte, aldosterone, and BUN levels, and blood pressure parameters, one-way ANOVA was used to compare groups. For NKCC2 and NKA abundance, comparisons were conducted using the mean control ratio as a standard for all experimental mean ratios. Ratios from all samples were analyzed using one-way ANOVA. Kruskal-Wallis one way analysis, followed by a post hoc test, was performed for histologic scores. TUNEL assay tubule and glomerular group percentage means were analyzed among experimental groups using one-way ANOVA.

For celecoxib concentration levels, data analysis was completed using a Student’s t-test. Statistical significance was conferred at p<0.05. Values are represented as mean ± standard error of the mean.

## Results

### Effect of Drugs on Body, Heart, and Kidney Weight

To determine drug effects on organ and body mass, animals were weighed on day 10 prior to euthanasia. The heart and kidneys were excised and weighed before experimental analysis. Animal body weight ranged from 240.67±5.71 to 260.60±6.79 (Table 1). There was no significant difference between total bodyweight of any of the treatment groups (p = 0.1785). Heart and kidney weights did not fluctuate to any significant degree by the end of the study (p = 0.4547 and p = 0.5618, respectively) and no significant alterations in heart to bodyweight or kidney to bodyweight ratios were noted (p = 0.7814 and p = 0.2073, respectively).

**Table 1:** Total body, kidney, and heart weight with organ to body ratio.

Group	Body (g)	Kidney (g)	Kidney/Body	Heart (g)	Heart/Body
**VEH+VEH**	250.10±5.23	0.88±0.06	0.0035±0.0002	0.90±0.04	0.0036±0.0001
**VEH+CEL**	244.00±8.04	0.79±0.05	0.0033±0.0002	0.91±0.03	0.0038±0.0002
**MISO+MISO**	240.67±5.71	0.89±0.07	0.0037±0.0002	0.93±0.03	0.0039±0.0001
**MISO+CEL**	260.60±6.79	0.82±0.05	0.0031±0.0002	1.00±0.07	0.0039±0.0004

VEH+VEH – vehicle+vehicle; VEH+CEL – vehicle+celecoxib; MISO+MISO – misoprostol+misoprostol; MISO+CEL – misoprostol+celecoxib.

The values were not significantly different, p>0.05.

### Effect of Drugs on Electrolyte Excretion Rates and Plasma Levels

**Figure 1:**
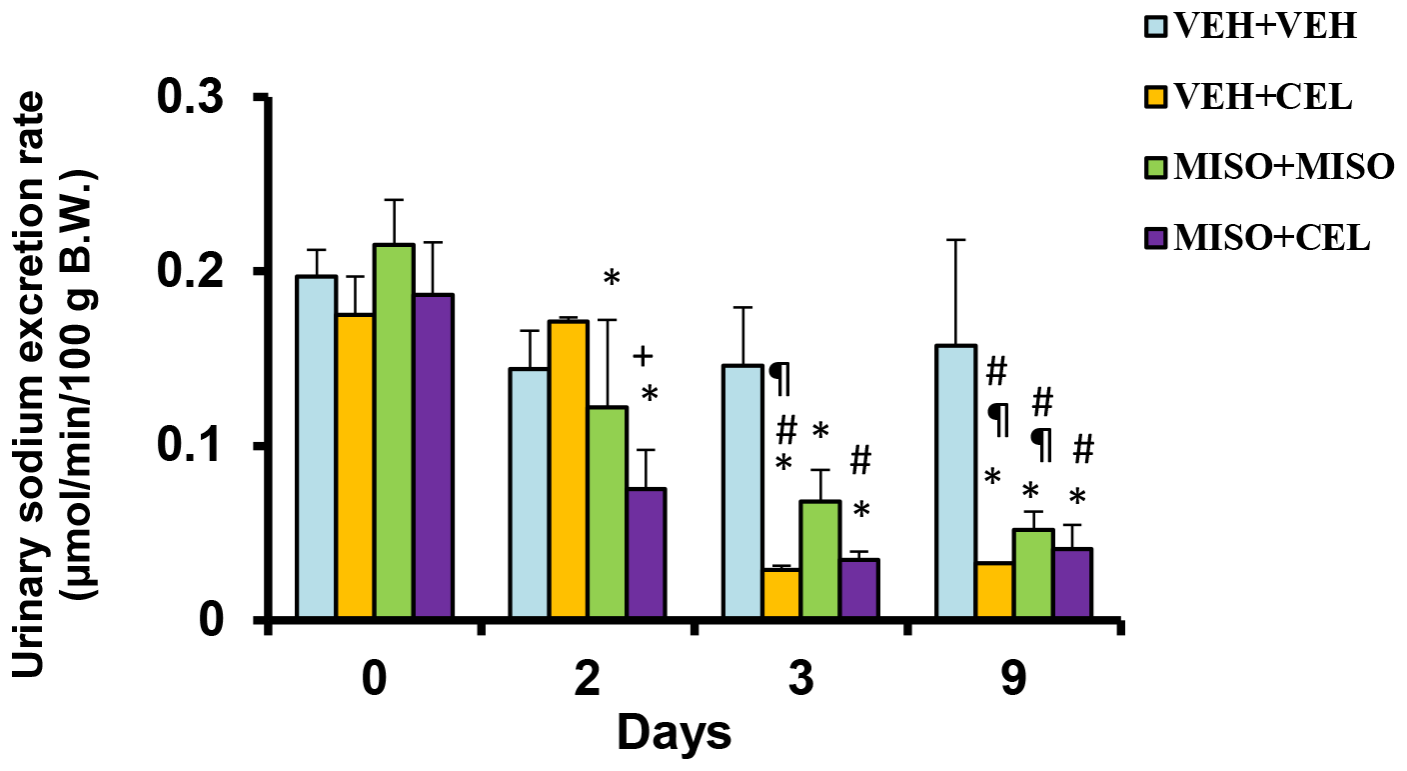
Effect of treatment with vehicle+vehicle (VEH+VEH), vehicle+celecoxib (VEH+CEL), misoprostol+misoprostol (MISO+MISO), or misoprostol+celecoxib (MISO+CEL) on sodium excretion rate. *p<0.05, significantly different from baseline. ^¶^p<0.05, significantly different from day 2. ^#^p<0.05, significantly different from VEH+VEH. ^+^p<0.05, comparison of VEH+CEL group with MISO+CEL group.

To determine drug effects on electrolyte excretion, urinary samples collected on days 0, 2, 3 and 9 and plasma samples collected on day 10 were analyzed with an EasyLyte electrolyte analyzer. Baseline values ranged from 0.18±0.01 µmol/min/100 g to 0.23±0.04 µmol/min/100 g (Figure 1). On day 2, urinary excretion of sodium was significantly reduced in the misoprostol+misoprostol (0.12±0.05 µmol/min/100 g, p = 0.0037) and misoprostol+celecoxib (0.07±0.02 µmol/min/100 g, p = 0.0006) groups when compared to baseline (0.22±0.03 µmol/min/100 g and 0.19±0.03 µmol/min/100 g, respectively). At day 3, all treatment groups began showing significant reductions in sodium excretion when compared to baseline. Groups receiving vehicle+celecoxib (0.03±0.002 µmol/min/100 g) or misoprostol+celecoxib (0.03±0.005 µmol/min/100 g) showed significant reduction in sodium excretion when compared to vehicle+vehicle (0.15±0.033 µmol/min/100 g, p<0.01). By day 9, all treatment groups showed significant reductions in sodium excretion compared to control.

**Figure 2:**
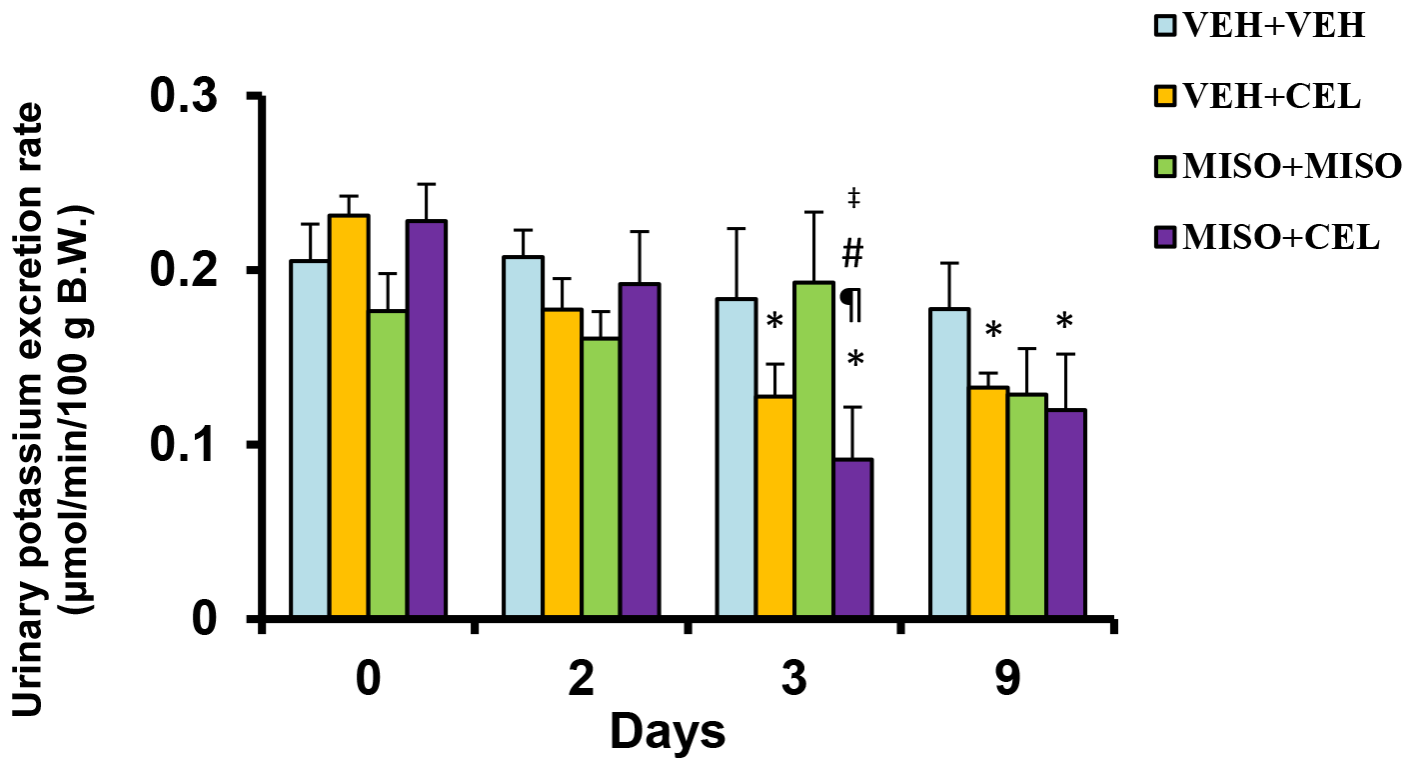
Effect of treatment with vehicle+vehicle (VEH+VEH), vehicle+celecoxib (VEH+CEL), misoprostol+misoprostol (MISO+MISO), or misoprostol+celecoxib (MISO+CEL) on potassium excretion rate. *p<0.05, significantly different from baseline. ^¶^p<0.05, significantly different from day 2. ^#^p<0.05, significantly different from VEH+VEH. ^‡^p<0.05, comparison of MISO+MISO group with MISO+CEL group.

Potassium excretion was significantly lower in vehicle+celecoxib and misoprostol+celecoxib groups when compared to baseline (Figure 2). At day 3, potassium excretion was significantly lower in the misoprostol+celecoxib group (0.09±0.03 µmol/min/100 g) when compared to both baseline (0.23±0.02 µmol/min/100 g, p = 0.0011), day 2 (0.19±0.03 µmol/min/100 g, p = 0.0140) and vehicle+vehicle (0.18±0.04 µmol/min/100 g, p = 0.0267) values. Potassium excretion was significantly reduced in comparison to baseline for both the vehicle+celecoxib group (0.23±0.01 µmol/min/100 g versus 0.13±0.01 µmol/min/100 g, p = 0.0456) and the misoprostol+celecoxib group (0.23±0.02 µmol/min/100 g versus 0.12±0.03 µmol/min/100 g, p = 0.0126) through day 9.

Comparisons of electrolyte plasma values showed no significant changes in sodium (p = 0.3128) or potassium (p = 0.3838) levels for any treatment group (Table 2).

**Table 2:** Sodium and potassium plasma levels on day 10.

Group	Sodium (mM)	Potassium (mM)
**VEH+VEH**	134.16±0.91	6.26±0.72
**VEH+CEL**	136.80±1.56	4.89±0.66
**MISO+MISO**	136.08±0.66	5.68±0.60
**MISO+CEL**	136.68±1.01	4.99±0.33

VEH+VEH – vehicle+vehicle; VEH+CEL – vehicle+celecoxib; MISO+MISO – misoprostol+misoprostol; MISO+CEL – misoprostol+celecoxib.

The values were not significantly different, p>0.05.

### Effect of Drugs on Urinary KIM-1 Concentrations

**Figure 3:**
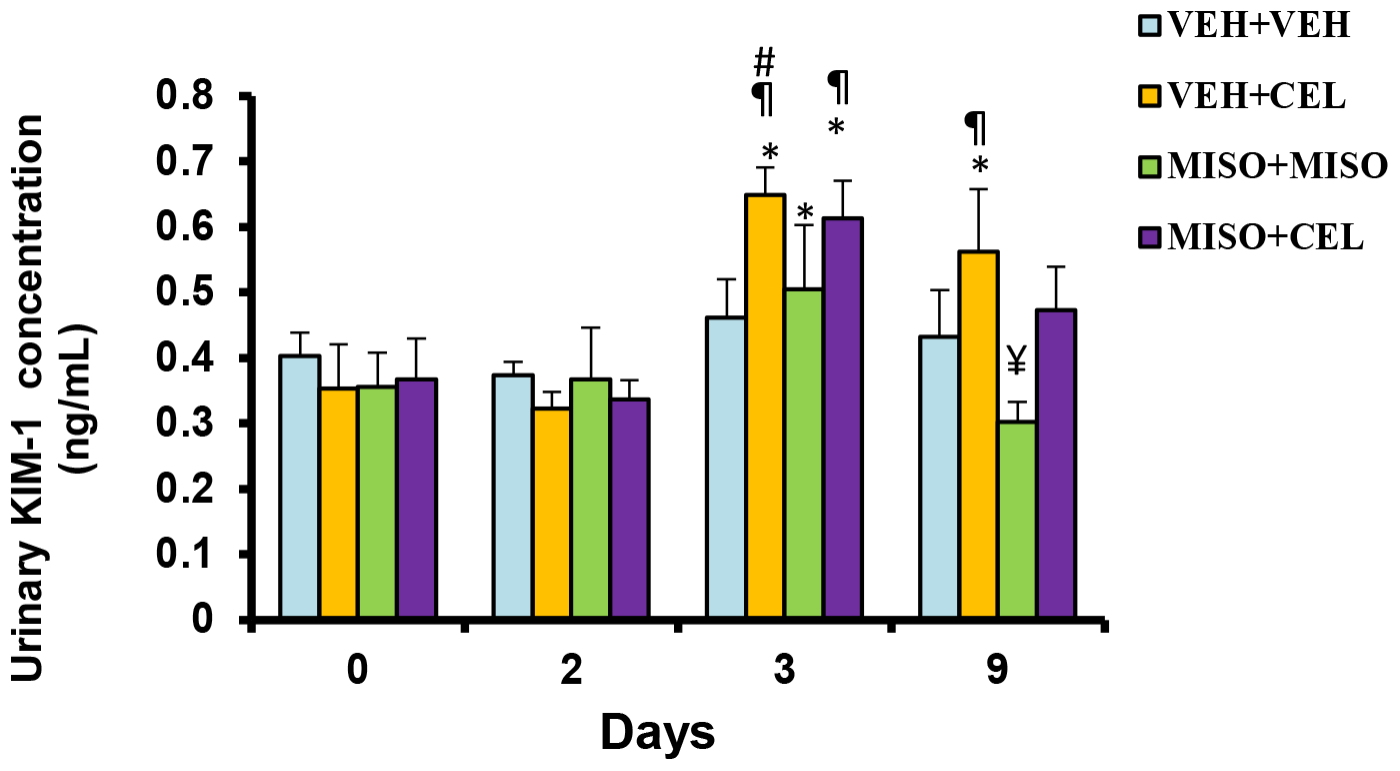
Effect of treatment with vehicle+vehicle vehicle (VEH+VEH), vehicle+celecoxib (VEH+CEL), misoprostol+misoprostol (MISO+MISO), or misoprostol+celecoxib (MISO+CEL) on KIM-1 concentration. *p<0.05, significantly different from baseline. ^¶^p<0.05, significantly different from day 2. ^#^p<0.05, significantly different from placebo. ^¥^p<0.05, significantly different from day 3.

As shown in Figure 3, baseline KIM-1 levels ranged from 0.35±0.07 to 0.38±0.05 ng/mL. Compared to the baseline values, a significant increase in KIM-1 levels was observed in both the vehicle+celecoxib group (0.65±0.02 versus 0.35±0.07 ng/mL, p = 0.0002) and misoprostol+celecoxib group (0.61±0.06 versus 0.37±0.06 ng/mL, p = 0.0015) on day 3. At day 9, vehicle+celecoxib (0.56±0.10) treated rats showed significantly increased KIM-1 expression compared to both baseline (0.35±0.07, p = 0.0106) and day 2 (0.32±0.02, p = 0.0036) values, while animals treated with misoprostol+misoprostol (0.30±0.03) show significantly reduced KIM-1 levels in comparison to day 3 (0.50±0.10, p = 0.0090). No significant difference was noted in KIM-1 levels between vehicle+celecoxib and misoprostol+celecoxib groups (p = 0.6624).

### Effect of Drugs on Aldosterone

**Figure 4:**
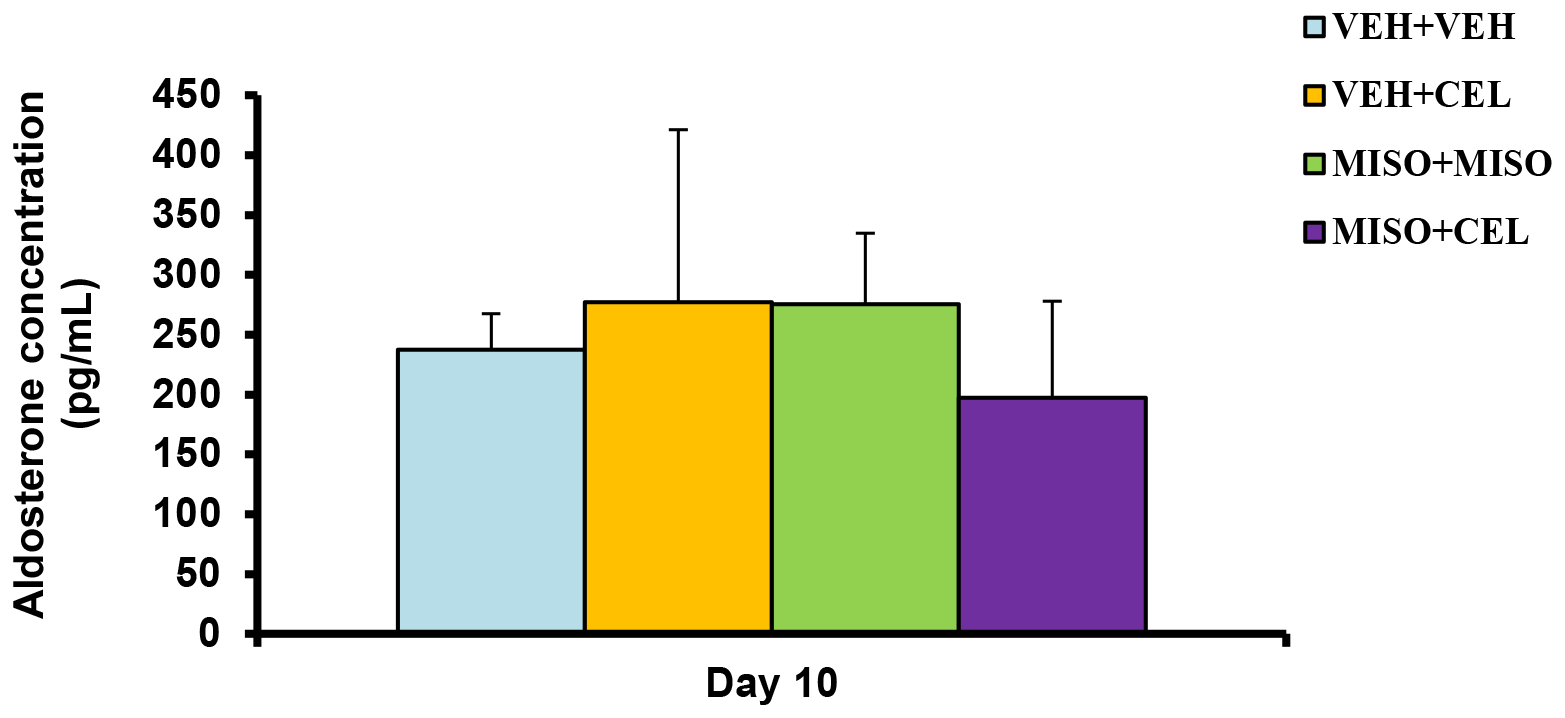
Effect of treatment with vehicle+vehicle (VEH+VEH), vehicle+celecoxib (VEH+CEL), misoprostol+misoprostol (MISO+MISO), or misoprostol+celecoxib (MISO+CEL) on aldosterone concentrations. The values were not significantly different, p>0.05.

To determine probable cause of electrolyte retention, circulating aldosterone levels on day 10 were measured using blood collected via heart puncture. As has been indicated in Figure 4, final day aldosterone plasma concentrations ranged from 197.49±80.59 to 277.26±143.93 pg/mL. No significant difference was noticed among the treatment groups (p = 0.8269).

### Effect of Drugs on BUN Concentration

**Figure 5:**
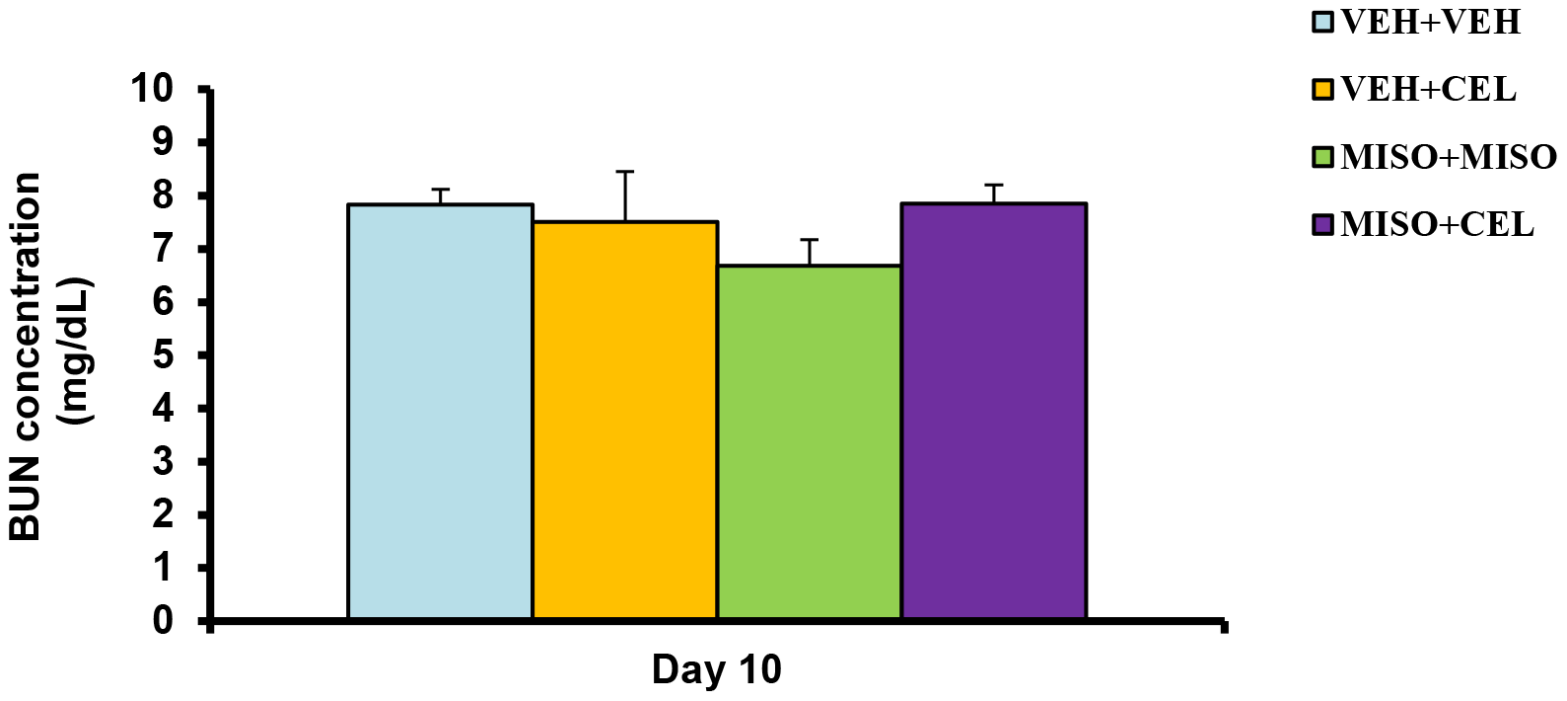
Effect of treatment with vehicle+vehicle (VEH+VEH), vehicle+celecoxib (VEH+CEL), misoprostol+misoprostol (MISO+MISO), or misoprostol+celecoxib (MISO+CEL) on BUN. The values were not significantly different, p>0.05.

To determine the extent of uremia within treatment groups, BUN levels on day 10 were measured using blood collected via heart puncture. Treatment of misoprostol, celecoxib or their combination did not increase BUN levels any appreciable degree among treatment groups (p = 0.4225) (Figure 5). Average BUN concentrations ranged from 7.85±0.35 to 6.68±0.49 mg/dl (Figure 5).

### Effect of Drugs on cTnI

**Figure 6:**
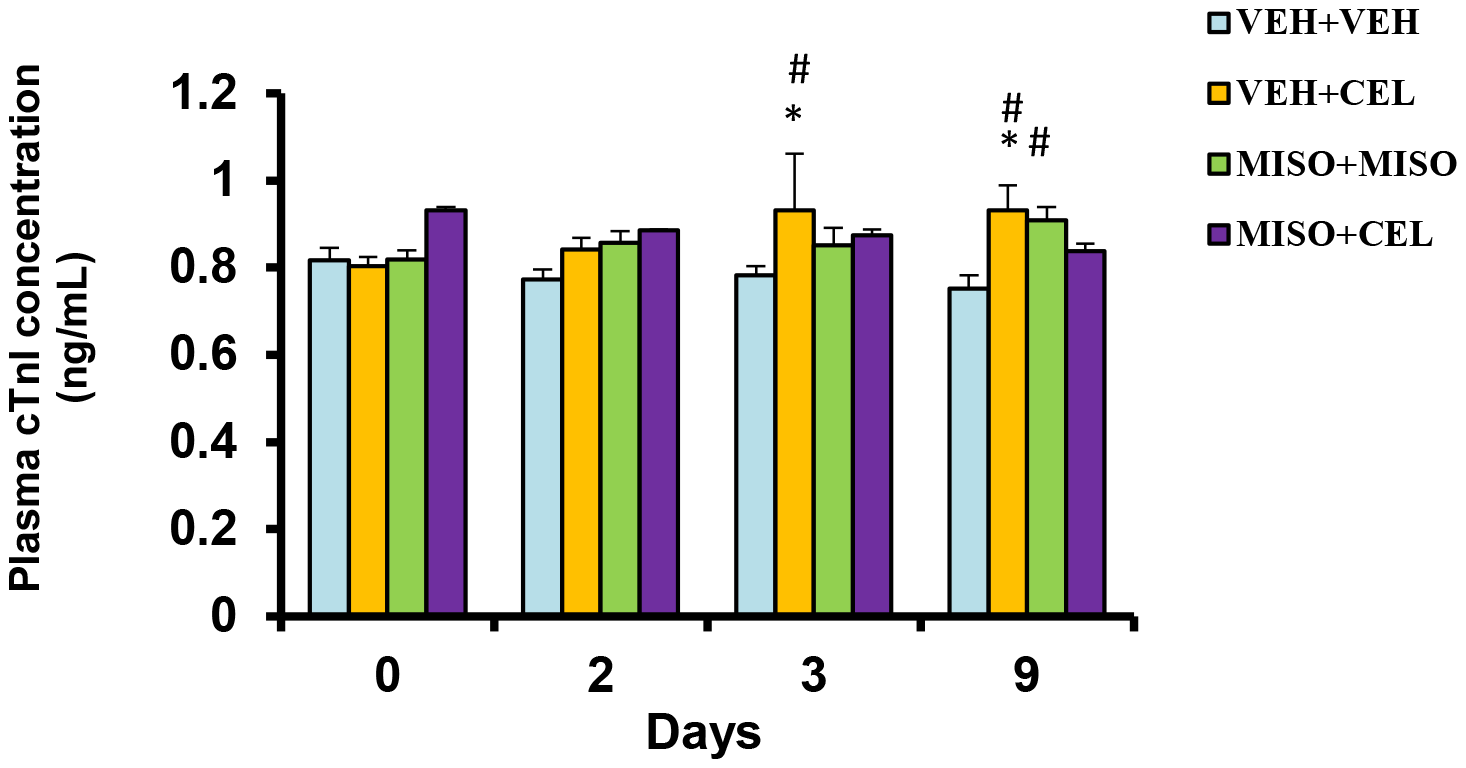
Effect of treatment with vehicle+vehicle (VEH+VEH), vehicle+celecoxib (VEH+CEL), misoprostol+ misoprostol (MISO+MISO), or misoprostol+celecoxib (MISO+CEL) on cTnI. *p<0.05, significantly different from baseline. ^#^p<0.05, significantly different from VEH+VEH.

To evaluate the effects of celecoxib and/or misoprostol on CV health, cTnI assays were performed to measure the extent of cTnI expression at baseline, day 2, day 3 and day 9 (Figure 6). On day 3, rats treated with vehicle+celecoxib had significant increases in cTnI expression when compared to baseline and vehicle+vehicle (p = 0.0467 and p = 0.0171, respectively). At day 9, vehicle+celecoxib treated groups maintained elevated cTnI levels in comparisons to baseline and vehicle+vehicle (p = 0.0203 and p = 0.0040, respectively); while the misoprostol+misoprostol group showed a significant elevation in cardiac cTnI expression when compared to vehicle+vehicle (p = 0.0078). No significant differences were noted among the treatment groups at any time point.

### Effect of Drugs on Blood Pressure Parameters

To measure the cardiac effects of celecoxib and misoprostol, blood pressure measurements were taken for each group (Table 3). On day 9, there were no significant differences in blood pressure parameters between the vehicle+celecoxib group and the vehicle+vehicle group. The misoprostol+misoprostol group showed significant increases in systolic (p = 0.0017) and mean blood pressure (p = 0.0304) when compared to the vehicle+vehicle group; while rats receiving misoprostol+celecoxib showed significant increases in diastolic (p = 0.0054), systolic (p = 0.0171), and mean blood pressure (p = 0.0036). When compared to vehicle+celecoxib, rats treated with misoprostol+celecoxib showed significant increases in diastolic and mean blood pressure (p = 0.0071 and p = 0.0153, respectively).

**Table 3:** Blood pressure parameters measured on day 9.

Group	Diastolic pressure (mmHg)	Systolic pressure (mmHg)	Mean pressure (mmHg)	Heart rate (beats/min)
**VEH+VEH**	92.35±2.44	141.13±1.79	108.12±2.02	427.96±9.31
**VEH+CEL**	91.64±3.27	144.90±3.40	109.03±3.14	448.26±15.00
**MISO+MISO**	98.41±3.28*	151.76±2.49*	115.89±2.84* ^#^	447.97±10.52
**MISO+CEL**	103.70±3.06* ^#+^	149.11±2.31*	118.55±2.67* ^#^	446.55±10.55

VEH+VEH – vehicle+vehicle; VEH+CEL – vehicle+celecoxib; MISO+MISO – misoprostol+misoprostol; MISO+CEL – misoprostol+celecoxib.

*p<0.05, significantly different from VEH+VEH.

# p<0.05, significantly different from VEH+CEL.

+ p<0.05, comparison of VEH+CEL group with MISO+CEL group.

### Histopathologic Assessment of the Kidney

**Figure 7:**
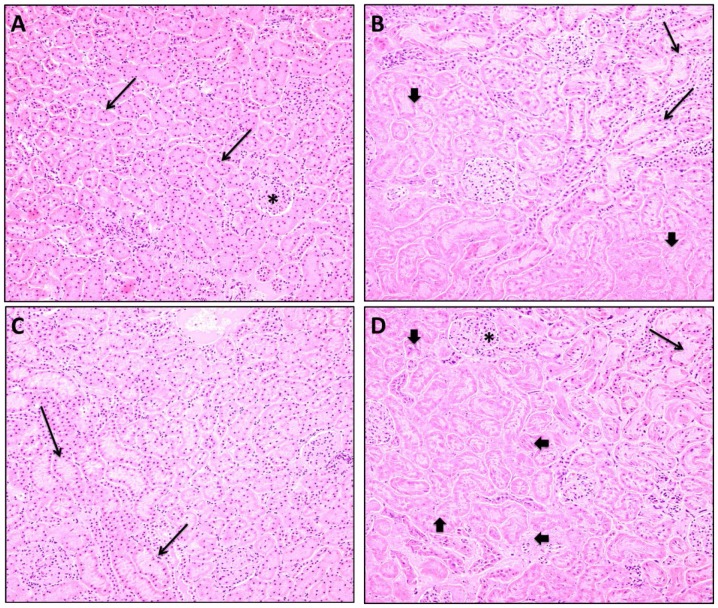
Kidney sections (40X) from vehicle+vehicle group showing glomeruli (*) and normal tubules (arrow) without dilation or necrosis. (A), vehicle+celecoxib group showing areas of moderate tubule necrosis (arrowhead) and mild tubule dilatation (arrow) (B), misoprostol+misoprostol group showing mild tubular dilatation (arrow) without necrosis (more normal tubules are seen to the right of the photomicrograph) (C), misoprostol+celecoxib group showing a large area of marked tubule necrosis (arrowhead) with relative sparing of the glomeruli (*), and moderate tubular dilation (arrow) (D).

In the kidney, no significant histopathological changes were noted in the glomeruli (Figure 7). However, groups receiving vehicle+celecoxib or misoprostol+celecoxib showed a significant increase in tubular necrosis compared to vehicle+vehicle (Mean Rank Score: vehicle+vehicle versus vehicle+celecoxib, 35.0 versus 67.0 (p<0.05); vehicle+vehicle versus misoprostol+celecoxib, 35.0 versus 70.0 (p<0.05) (Table 4). All groups receiving treatment showed significant increases in tubular dilatation; however, dilatation was more severe in the group treated with misoprostol+celecoxib compared to the vehicle+celecoxib group (Mean Rank Score: vehicle+celecoxib versus misoprostol+celecoxib; 61.5 versus 78.0 (p<0.05).

**Table 4:** Assessment of tubular necrosis and dilatation.

	Tubular Necrosis Score						Tubular Dilatation Score					
Group	0	1	2	3	n	Mean-Rank	0	1	2	3	n	Mean-Rank
**VEH+VEH**	5	0	0	0	**5**	**35.0**	4	1	1	0	**6**	**45.0**
**VEH+CEL**	3	2	1	0	**6**	**67.0** #	2	3	1	0	**6**	**61.5** #
**MISO+MISO**	5	0	0	0	**5**	**35.0**	0	5	0	0	**5**	**69.0** #
**MISO+CEL**	0	0	2	2	**4**	**70.0** ^#^ ‡	0	0	4	0	**4**	**78.0** ^+#^

VEH+VEH – vehicle+vehicle; VEH+CEL – vehicle+celecoxib; MISO+MISO – misoprostol+misoprostol; MISO+CEL – misoprostol+celecoxib.

# p<0.05, significantly different from VEH+VEH.

‡ p<0.05, comparison of MISO+MISO group with MISO+CEL group.

+ p<0.05, comparison of VEH+CEL group with MISO+CEL group.

### Histopathologic Assessment of the Heart

**Figure 8:**
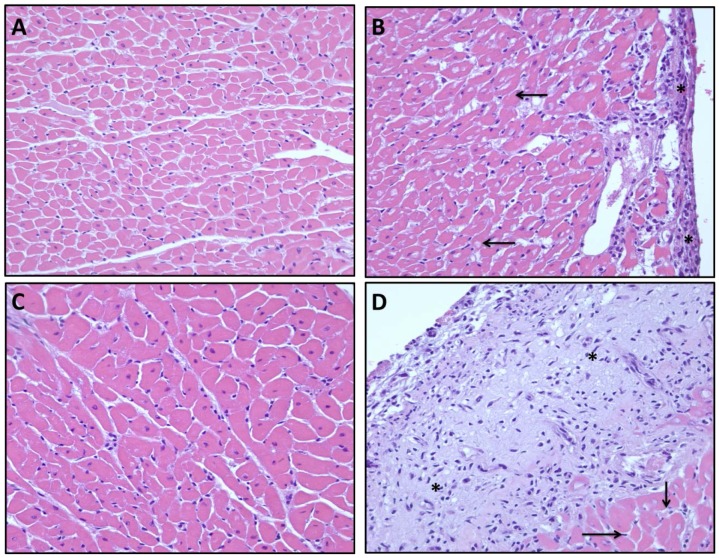
Cross section (40X) of normal cardiac myocytes from vehicle+vehicle group. (A), vehicle+celecoxib group showing a mild organizing pericarditis (*) and adjacent normal cardiac myocytes (arrow) (B), normal cardiac myocytes from misoprostol+misoprostol group (C), misoprostol+celecoxib group showing a severe organizing pericarditis (*), and adjacent normal cardiac myocytes (arrow) (D).

In general, no significant histopathological changes were noted in the myocardium (Figure 8). Three animals, one in the vehicle+celecoxib group and two in misoprostol+celecoxib group, exhibited a significant organizing pericarditis. However, no inflammation or changes of ischemia, infarct or myocardial injury was seen.

### TUNEL Assay

**Figure 9:**
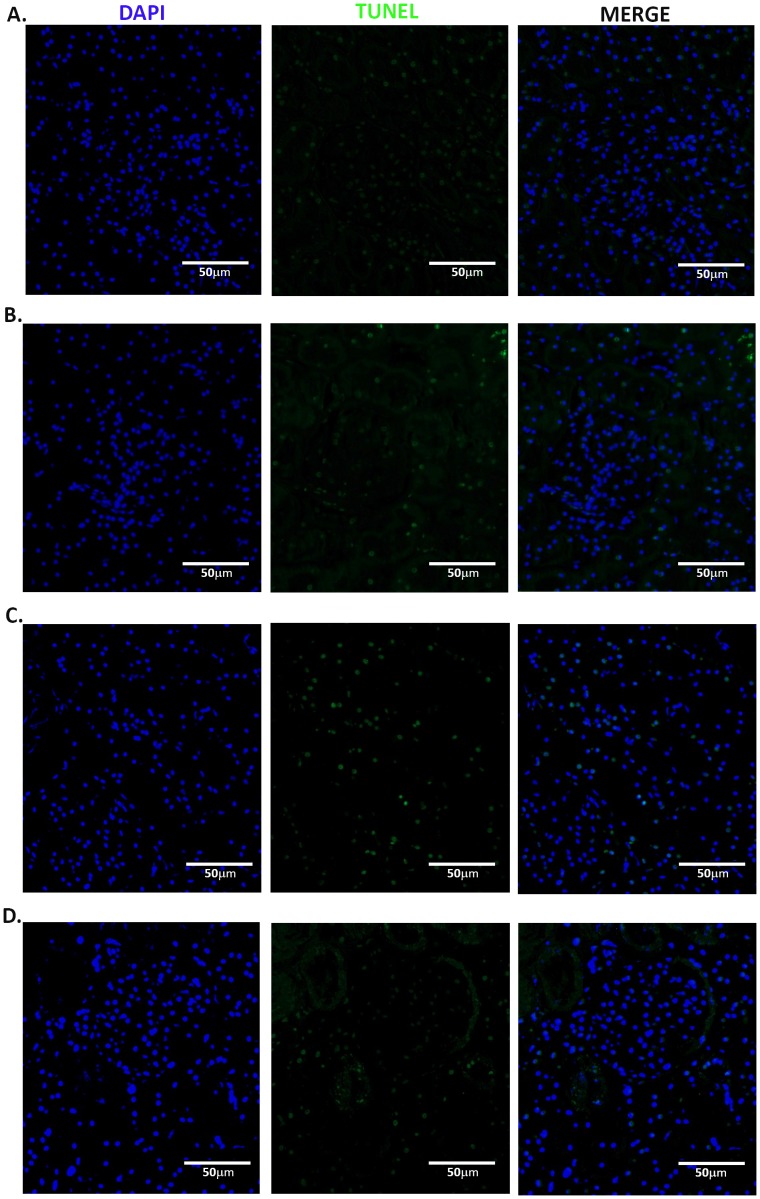
Glomerular TUNEL assay consisting of DAPI (nuclei), fluorescein (apoptotic marker), and merged sections for each group (vehicle+vehicle (A); vehicle+celecoxib (B); misoprostol+misoprostol (C); misoprostol+celecoxib (D)). 20x magnification.

**Figure 10:**
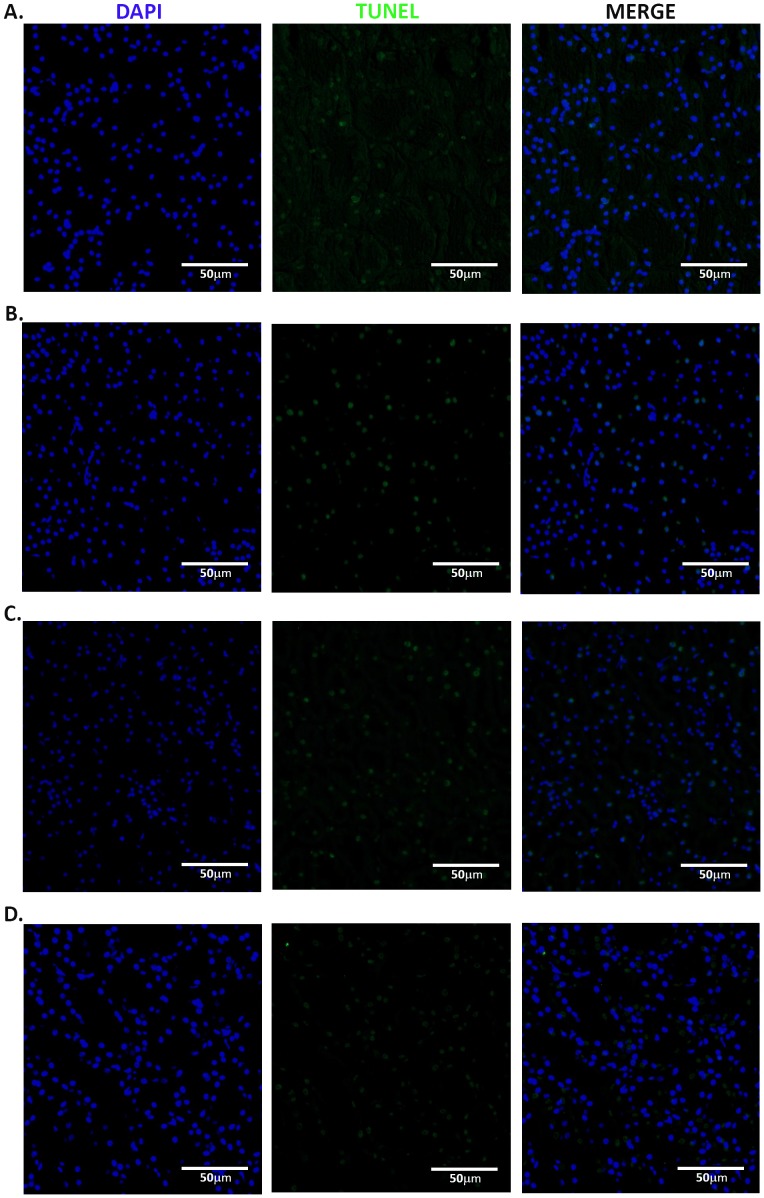
Tubular TUNEL assay consisting of DAPI (nuclei), fluorescein (apoptotic marker), and merged sections for each group (vehicle+vehicle (A); vehicle+celecoxib (B); misoprostol+misoprostol (C); misoprostol+celecoxib (D)), 20x magnification.

Glomerular and tubular TUNEL analysis are presented visually in Figures 9 & 10, respectively. As seen in Table 5, the administration of vehicle+celecoxib, misoprostol+misoprostol, or misoprostol+celecoxib does not significantly alter tubular nor glomerular apoptotic levels (p = 0.144 and p = 0.485, respectively).

**Table 5:** Quantification of apoptosis levels within glomeruli and tubules.

Group	Glomeruli	Tubules
**VEH+VEH**	31.79±2.26	28.21±1.99
**VEH+CEL**	33.82±3.31	21.94±3.09
**MISO+MISO**	30.48±2.57	20.12±1.95
**MISO+CEL**	36.91±3.62	25.96±3.22

VEH+VEH – vehicle+vehicle; VEH+CEL – vehicle+celecoxib; MISO+MISO – misoprostol+misoprostol; MISO+CEL – misoprostol+celecoxib. Values presented as mean apoptotic cell percentage ± SEM.

The values were not significantly different, p>0.05.

### Immunohistochemical Analysis of Caspase-3

**Figure 11:**
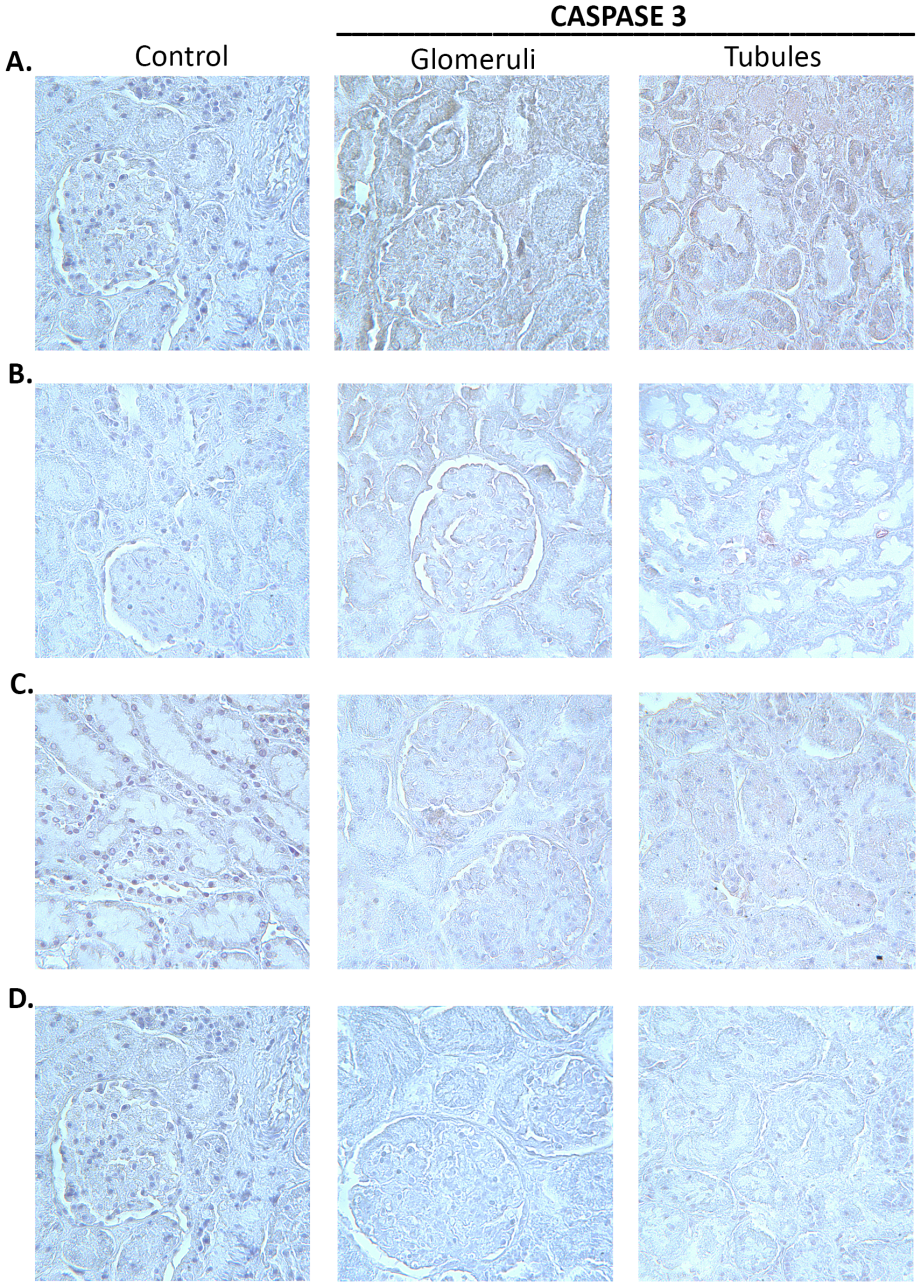
Caspase-3 immunohistochemistry consisting of an isotype control, glomerular, and tubular sections for each group (vehicle+vehicle (A); vehicle+celecoxib (B); misoprostol+misoprostol (C); misoprostol+celecoxib (D)), 40x magnification.

Figure 11 shows Caspase-3 immunohistochemical labeling. There does not appear to be a significant difference in Caspase-3 abundance between treatment groups compared to control neither within glomeruli nor tubules.

### Western Blot Analysis of NKCC2 Abundance

**Figure 12:**
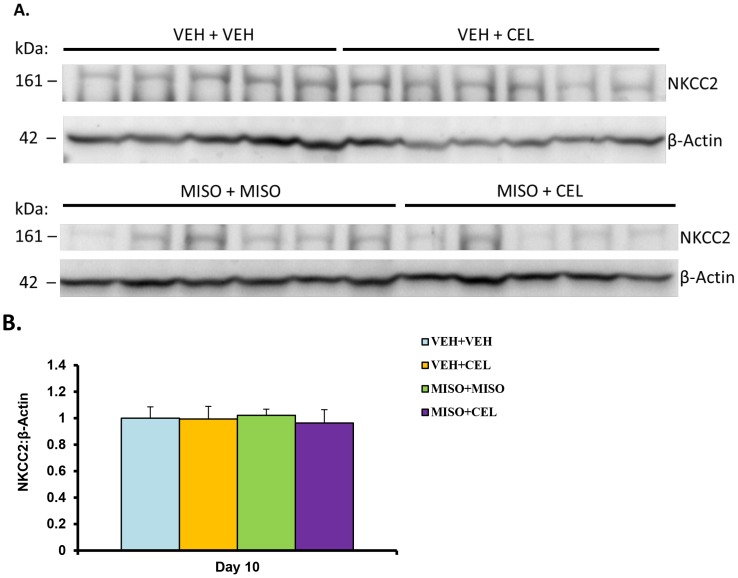
NKCC2 expression (A) and normalized bands (B) in treatment with vehicle (VEH+VEH), vehicle+celecoxib (VEH+CEL), misoprostol+misoprostol (MISO+MISO), or misoprostol+celecoxib (MISO+CEL). Each immunoblot was conducted in triplicate. The values were not significantly different, p>0.05.

Figure 12A displays data collected from immunoblotting of renal NKCC2 and β-Actin. Western blot examination of whole kidney for NKCC2 abundance revealed no significant difference among treatment groups normalized to the control NKCC2: β-Actin ratio (normalize band densities: control, 1.00±0.09; vehicle+celecoxib, 0.99±0.09; misoprostol+misoprostol, 1.02±0.05; misoprostol+celecoxib, 0.96±0.10; p = 0.9910) (see Figure 12B). Thus administration of vehicle+celecoxib, misoprostol+misoprostol, or misoprostol+celecoxib did not significantly influence the levels of NKCC2 in these animals.

### Immunohistochemical Analysis of NKCC2

**Figure 13:**
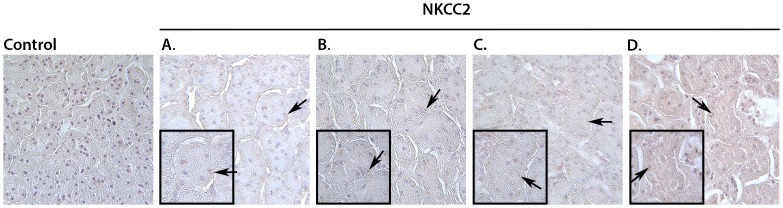
NKCC2 immunohistochemistry consisting of an isotype control, then one section from each group (vehicle+vehicle (A); vehicle+celecoxib (B); misoprostol+misoprostol (C); misoprostol+celecoxib (D)) showing the presence of NKCC2 (arrows). 40x magnification and insert at 100x.

Immunohistochemically labeled NKCC2 is shown in Figure 13. Compared to vehicle+vehicle, NKCC2 abundance does not appear to change within experimental groups.

### Western Blot Analysis of NKA Abundance

**Figure 14:**
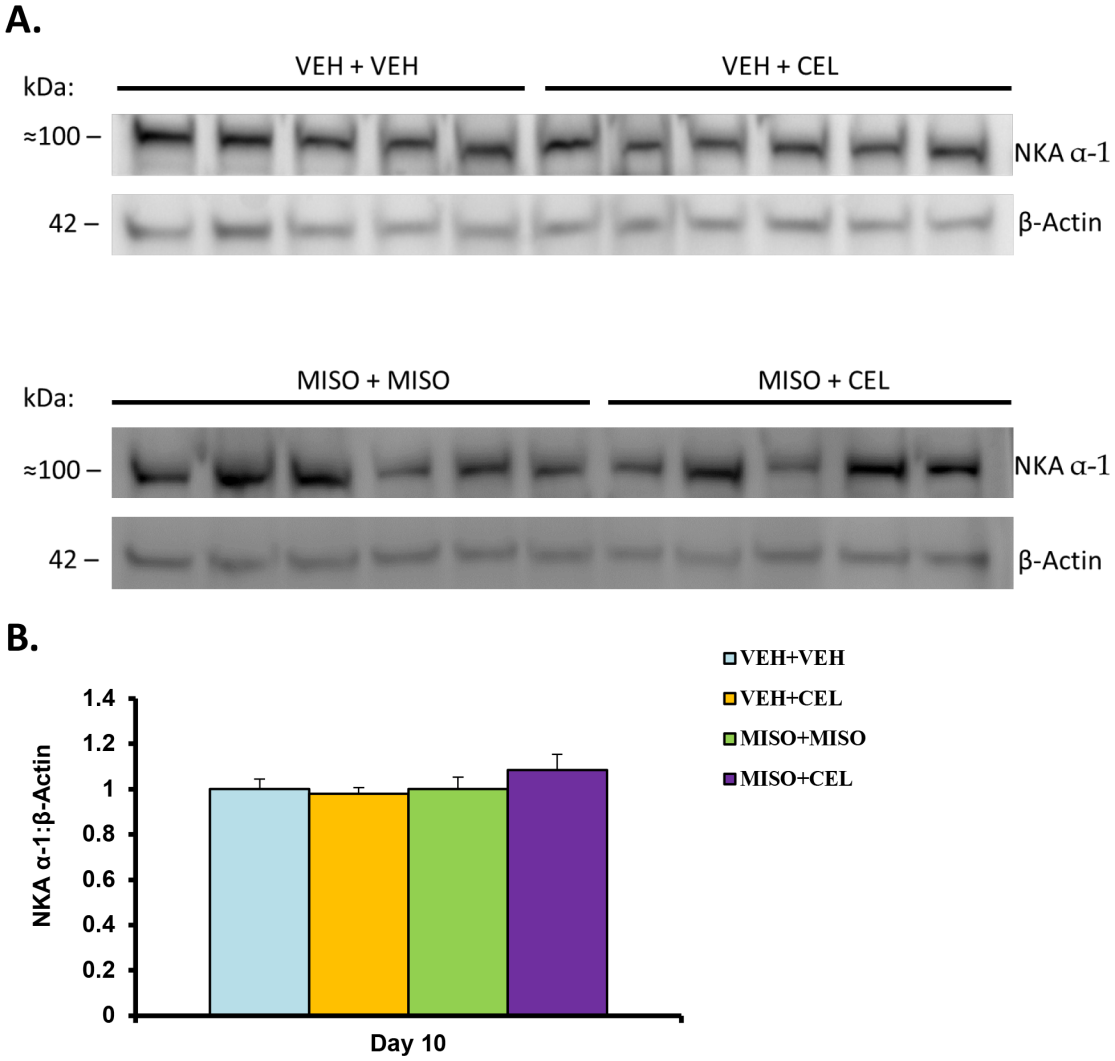
NKA α-1 expression (A) and normalized bands (B) in treatment with vehicle (VEH+VEH), vehicle+celecoxib (VEH+CEL), misoprostol+misoprostol (MISO+MISO), or misoprostol+celecoxib (MISO+CEL). Each immunoblot was conducted in triplicate. The values were not significantly different, p>0.05.

Figure 14A displays data collected from immunoblotting of renal NKA and β-Actin. Western blot examination of whole kidney for NKA abundance revealed no significant difference among treatment groups normalized to the control NKA α-1: β-Actin ratio (normalize band densities: control, 1.00±0.05; vehicle+celecoxib, 0.98±0.03; misoprostol+misoprostol, 1.00±0.05; misoprostol+celecoxib, 1.09±0.0; p = 0.446) (see Figure 14B). Thus administration of vehicle+celecoxib, misoprostol+misoprostol, or misoprostol+celecoxib did not significantly influence the levels of NKA in these animals.

### Levels of Celecoxib in Presence or Absence of Misoprostol

Plasma, kidney, and heart samples were analyzed to determine if misoprostol would alter celecoxib blood and tissue concentrations in the misoprostol+celecoxib group (Table 6). In plasma, drug concentrations of celecoxib in the vehicle+celecoxib group were not significantly different from those of the misoprostol+celecoxib group (p = 0.4710). Kidney celecoxib concentrations in vehicle+celecoxib group were not significantly different from misoprostol+celecoxib group (p = 0.9198). Analysis of the heart tissue showed no significant alterations in celecoxib concentration between the vehicle+celecoxib and misoprostol+celecoxib groups (p = 0.1446).

**Table 6:** Celecoxib concentration in the plasma, kidney, and heart.

	Celecoxib Concentration		
Group	Plasma (µg/mL)	Kidney (µg/g)	Heart (µg/g)
**VEH+CEL**	3.29±0.78	4.66±1.47	2.76±1.20
**MISO+CEL**	2.50±0.70	5.00±3.18	0.66±0.32

VEH+CEL – vehicle+celecoxib; MISO+CEL – misoprostol+celecoxib.

The values were not significantly different, p>0.05.

## Discussion

NSAIDs exert their side effects (e.g. GI, renal) by reducing the production of PGs. Celecoxib is a potent anti-inflammatory drug that functions by selectively inhibiting the COX-2 enzyme and subsequently reducing PG synthesis [11], [35].

Misoprostol, a PG-based medicine, has been used for prevention of NSAID-induced gastric injury. Misoprostol functions as a PGE_1_ analogue that offsets the deleterious effects of NSAIDs related to a reduction in PG biosynthesis and has been effectively used to offset gastric-related side effects often associated with NSAID consumption [24], [36]–[39].

The deleterious effects of NSAIDs on renal and CV systems have been well documented and extensively studied over the years. Interestingly, there have also been several studies that report the beneficial and protective effects of misoprostol consumption on CV disease formation, renal damage, and nephrotoxicity [30], [40], [41]. However, few studies have analyzed the CV and renal effects of concomitant administration of misoprostol with NSAIDs. Although the CV effects of select NSAIDs have been well documented [8], [42], the current literature has reported conflicting evidence in regard to the CV effects of celecoxib [42]. This study was designed to investigate the influence of concomitant administration of misoprostol on renal adverse effects of celecoxib. In addition, we studied the effect of celecoxib alone or in combination with misoprostol on some CV parameters in rats.

Renal injury is known to initially manifest through a reduction in electrolyte excretion and total urine outflow [43]. In past studies, misoprostol has been shown to delay the nephrotoxic effects of NSAIDs in cirrhotic patients and maintain normal urinary excretion and electrolyte output [44]–[46]. In our present study, we investigated the effect of misoprostol on celecoxib induced renal impairment by measuring and comparing total electrolyte excretion between treatment groups. Misoprostol given at a dose of 200 µg/kg daily did not attenuate sodium retention when given simultaneously with celecoxib (Figure 1). Likewise, the rate of potassium retention was also unaffected by the addition of misoprostol (Figure 2). These findings suggest that misoprostol was unable to attenuate NSAID-induced electrolyte retention. Interestingly, no significant change in plasma electrolyte concentrations was observed among the treatment groups. These findings are reflective of the compensatory change in body hemodynamics brought forth by altered renal health. Various hormones such as aldosterone, renin, and angiotensin act to control and mediate the balance between total body electrolyte concentrations and plasma volume. However, as shown in Figure 4, aldosterone levels on day 10 were not significantly altered among experimental groups compared to control. Thus electrolyte balance is being maintained through another mechanism. Balance may be maintained through vasopressin and anti-diuretic hormone (ADH) alterations. When plasma becomes concentrated, ADH is secreted in response to increased plasma osmolarity [47]. Previous studies have shown that ADH release is controlled by osmatic stimuli brought forth from changes in total electrolyte balance [47], [48]. It is entirely possible that the results of our study, showing normalized plasma electrolyte balance, is a result of alterations in hemodynamic pathways involving ADH and other hormone cascades. The absence of a significant change in BUN (Figure 5) in this experiment support previous studies which indicate that while nonselective NSAIDs produce sodium retention and a reduced glomerular filtration rate (GFR), COXIBs do not influence GFR [49].

KIM-1 is a sensitive and robust urinary biomarker of acute renal injury [31]. KIM-1 is a class 1 transmembrane cellular glycoprotein expressed by proximal tubule epithelial cells in the presence of injury or ischemia, including injury of drug origin [50], [51]. Recent studies have shown a direct relationship between KIM-1 expression and symptomatic heart failure in patient populations. In one study, KIM-1 expression was increased in patients diagnosed with chronic heart failure [52]. Another study confirmed that an increase in KIM-1 levels predisposes elderly men towards an increased risk of heart failure [53]. Lekawanvijit *et al.* also demonstrated a cardiorenal relationship for this biomarker, as evidenced by significant post-MI increases in KIM-1 expression in a rat model [54]. In our study, we looked at the possible attenuating effects of misoprostol when given in conjunction with NSAIDs on KIM-1 expression. As shown in Figure 3, concomitant administration of misoprostol with celecoxib did not attenuate NSAID induced KIM-1 expression. These findings suggest that misoprostol does not exhibit renal protection in the presence of celecoxib.

Troponin is a protein complex found in striated muscle, including cardiac muscle. cTnI and cardiac troponin T (cTnT) are indicators of cardiac muscle damage in humans [55], and have also been useful in detecting cardiac damage in rats [56], [57]. In our study, we found significant increases in cTnI expression in groups treated with celecoxib on day 3 when compared to baseline (Figure 6). These levels remained elevated through day 9. Misoprostol co-administration did not attenuate the elevation of cTnI expression to any significant degree. Interestingly, when rat blood pressure was measured on day 9, there was a significant rise in diastolic, systolic, and mean pressure in the misoprostol+misoprostol group when compared to vehicle+vehicle group (Table 3). The influence of misoprostol on blood pressure can be explained by the renal vasoconstriction effect of misoprostol [58]. Furthermore, when misoprostol was co-administered with celecoxib, these blood pressure parameters were further elevated. Diastolic and mean blood pressure were significantly elevated in the misoprostol+celecoxib group compared to both the vehicle+vehicle and vehicle+celecoxib groups. These findings suggest that celecoxib induces CV stress as evidenced by a rise in cTnI levels and blood pressure parameters. Our findings also suggest that misoprostol does not exhibit any cardioprotective effects in the presence of celecoxib, but may exacerbate celecoxib associated CV side effects.

Renal histopathological analysis provided insight into structural changes effected by drug administration. The relative sparing of glomeruli suggests that primary filtration is unaffected by celecoxib and changes in ion exchange likely occur at reuptake across the renal tubules (Figure 7). As previously reported, administration of celecoxib produces tubular necrosis and dilatation [59]. Concomitant misoprostol and celecoxib administration was discovered to exacerbate tubular necrosis. Similar damage intensification has been shown in a previous study in which celecoxib plus paracetamol worsened renal damage compared to celecoxib alone [60]. However, renal celecoxib levels were not increased in the presence of misoprostol. Thus, tubular damage does not appear to be directly correlated with renal celecoxib concentration. The renal adverse effect intensifying property of misoprostol will need to be weighed against the gastroprotective property conferred through concomitant administration, especially in cases of prior renal dysfunction.

Because misoprostol administration has presented with strong anti-apoptotic effects, renal apoptosis was quantified to account for renal damage [22]. As the TUNEL assay revealed no significant change in glomeruli (Figure 9) nor tubules (Figure 10), the renal toxicity observed in this study seems to occur primarily through necrotic cell death. The results of Caspase-3 immunohistochemistry (Figure 11) serve as a confirmation of the TUNEL results, as seemingly no change in abundance occurred between control and experimental groups.

In this study, rats presenting with pericarditis were grouped together and compared to all other celecoxib receiving rats (Figure 8). A student’s t test of the two groups, pericarditis positive and negative, revealed no significant difference in celecoxib concentration between the groups (data not shown). As such, an alteration in celecoxib concentration does not appear to be responsible for the appearance of pericarditis. In humans, pericardial inflammation can arise as a result of drug induced damage, bacterial or viral infections, or MI; however, pericarditis may also be idiopathic in origin [61]. A review of current literature shows no previous articles which have reported pericarditis associated with NSAID administration.

Sodium retention functions as a hallmark of NSAID-induced renal dysfunction [62]. PGE_2_ reduces sodium reabsorption in the thick ascending limb of the loop of Henle [63] via an activity reduction in NKCC2 [64]. In the absence of PGE_2_, through inhibition by NSAIDs, the presence of NKCC2 is increased; however, misoprostol administration has been shown to reduce the amount of NKCC2 [26]. The absence of a significant NKCC2 abundance change in this study (Figure 12) is supported by the antagonistic effects suggested by Fernandez-Llama et al [26]. In addition, the outcome of NKCC2 immunohistochemistry analysis (Figure 13) was in line with our western blotting results. These findings suggest that the particular electrolyte excretion change is orchestrated via another mechanism. In the absence of an increase in NKCC2 abundance, NKA was examined as a possible driver of sodium retention [13], [14]. No significant change in NKA protein levels was detected in this experiment (Figure 14); however a change in transporter activity, not examined in this study, could act as a mechanism of sodium retention [13]. In previous studies, when given concomitantly with other drugs misoprostol has been shown to increase drug plasma concentrations significantly [65]. To test the possibility of drug-drug interactions between celecoxib and misoprostol, we examined drug concentrations of celecoxib in plasma and tissue samples. In this study, there was no significant difference in celecoxib concentrations between groups treated with vehicle+celecoxib as compared to groups treated with misoprostol+celecoxib (Table 6). These results indicate that plasma concentrations of celecoxib were not altered in the presence of misoprostol and that findings within our study were not a result of pharmacokinetic drug interaction between celecoxib and misoprostol.

There are several limitations to our study. We examined the cardiorenal side effects of celecoxib using a single daily dose of 40 mg/kg/day. At this dosage range we found significant correlation between electrolyte excretion, kidney and cardiac biomarker expression, and tissue necrosis in groups treated with celecoxib or misoprostol+celecoxib. However, the observed association between treatment groups and the accompanying side effects may not be conclusive since it was made using a single daily dose of our selected NSAID. Therefore, it is still unclear as to whether these effects where reached at the ascending or plateau stage of the drug exposure response curve. It is also important to note that our study focused extensively on NSAID-induced cardiorenal side effects in conjunction with misoprostol administration. The action of NSAIDs and misoprostol on NSAID-induced GI related events has been well documented [66]–[68]. As such, GI side effects were not elucidated within the context of this study. Finally, although electrolyte excretion rates were measured, dietary intake of electrolytes was not measured in this study. Thus changes in excretion may result from differences in food intake elicited by drug side effects.

In conclusion, concomitant administration of misoprostol with celecoxib did not alter reduced electrolyte excretion induced by celecoxib in this study. No significant difference was noticed between the combined treatment of misoprostol and celecoxib on KIM-1 expression. Similarly, histopathological evaluation of kidney tissue also confirmed NSAID-induced tubular necrosis in both treatment groups. Interestingly, celecoxib-treated groups showed significantly elevated cTnI levels, suggesting celecoxib induced cardiac stress. Misoprostol did not lessen cardiac stress to any significant degree and actually showed increased cTnI levels when compared to control. These findings suggest that misoprostol does not attenuate NSAID-induced kidney damage and may exacerbate cardiac stress. As such, further studies are warranted to understand the role misoprostol and celecoxib may play in the onset of cardiorenal events. The results obtained within this study suggest that more research should be conducted in patients who receive misoprostol and/or celecoxib therapy to examine for signs of CV and renal related issues.
